# Hope in a time of civicide: regenerative development and IPAT

**DOI:** 10.1186/s42055-020-00034-1

**Published:** 2020-08-24

**Authors:** Peter Newman

**Affiliations:** John Curtin Distinguished Professor of Sustainability, Curtin University Sustainability Policy (CUSP) Institute, School of Design and the Built Environment, Curtin University, Perth, Australia

## Abstract

This paper is  written as the world faces economic recovery after the Covid pandemic collapse. It also responds to the article in *Sustainable Earth* by Peter Hancock ‘In Praise of Civicide’ by creating a more hopeful vision of the future. Peter suggests the only hope is in psychological mind-sets that can change behaviour as nothing else will stop the path to destruction from present growth in population, the economy and technology. Rather than seeing inevitable civicidal elements, as devised in the IPAT model used by Peter and many others to explain global environmental destruction, the paper reassesses the fundamentals of this model developed by Paul and Anne Ehrlich in the 1960's. As the global economy has collapsed and environmental impacts improved everywhere, the Hancock argument based on IPAT would seem to have support. The paper shows how it is possible to grow again in the three IPAT factors if the world moves beyond sustainable development which just minimises impact to regenerative development which reclaims environmental impacts. If all three elements combine to create uncontrolled growth as was happening in the 60’s to 80’s then civicide is inevitable, but not if they change to regenerative development. The three stages of exploitive, sustainable and regenerative development turn IPAT from being negative to positive about civilization. These choices are very stark in the 2020’s. The technological possibilities of a regenerative future are outlined and the fundamentals needed for a sustainable earth are sketched, providing some evidence of hope for using the present pandemic and economic collapse as the basis for regenerating civilization not praising civicide.

## Introduction

I am writing this paper as the COVID-19 virus wreaks havoc across the earth, closing down airlines, businesses, public and private meetings, and generating violent scenes at supermarkets. The responses indicate how fragile is civilization and how rapidly it can collapse [5]. The death of thousands of people and the shut-down in economic activity are welcomed by some as they suggest that such interventions by natural processes will reduce global environmental impact, especially from fossil fuels whose prices have crashed through the floor in record declines, because population is being reduced and because, if the economy is the problem, then its slowing must be good. As Peter Hancock suggests in *Sustainable Earth,* perhaps we should be ‘In Praise of Civicide’ [14].

Civilization is certainly under threat in new ways as the 2020’s has opened with the astonishing and unprecedented Australian bushfires and then the pandemic. Both are challenging our perceptions of normal life as nature responds to growing human impacts [38]. However, is the welcoming of reduced population and declining economic growth the only real solution to our critical global environmental problems?

Peter Hancock’s *Sustainable Earth* Debate paper suggests that we are on an ‘evident path to destruction’ due to rapid population growth and economic growth that enables constantly increasing affluent consumption of resources; he is also cynical about technology’s ability to make the necessary changes like renewables and recycling as it is inevitably out of the control of those who want to make such changes. His solution is to suggest a ‘collective psychological mindset’ through cybernetics that shifts our behaviour. I will not be debating this behaviour question except to say there has been a remarkable behaviour change process underway in the early months of 2020 that could perhaps be a model of what he was suggesting. However, he dismisses the three factors of population, economic growth and technology (the IPAT model) using rather old data on trends that should now be re-examined, and as these factors are at the heart of civilization, this dismissal is why hope is a rather bleak option only in his paper.

There are many other writers who have understood this to be the only way the big issues of global environmental impact, summarised by the term Anthropocene, could be imagined as being solved. This has been a dominant approach since the days of *The Population Bomb* [7] and are popularised in other work such as Jackson [19]. In this perspective the earth will continue to suffer as long as these three factors are still growing. This is the IPAT model and I want to deconstruct it.

After describing how the IPAT model works, an approach will be shown that demonstrates how the inevitability of such a model can be challenged so that civilization becomes built around solving the global environmental issues as part of the economy and population growth; however, if this does not happen civicide is inevitable. Evidence will be given that change is happening. Finally, the paper will set out a vision of how emerging sustainability-based regenerative technology and associated socio-technical systems, can be mainstreamed and how the opportunity should now be taken to leap forward into regenerating the earth.

## The IPAT model

### IPAT unpacked

IPAT was developed by Paul and Anne Ehrlich 50 years ago [7, 8]. It is a system for understanding impact based on the interaction between the three factors of population P, affluence A, and technology T. It uses the equation:

Impact = Population x Affluence per capita (measured by GDP/person) x Technology per unit of Impact (measured by various impacts like carbon intensity).

I worked with the Ehrlich’s at Stanford in the early 1970’s and was heavily influenced by this model, teaching it for many years and writing my first environmental paper showing how the model could be applied at a national level [22, 23]. IPAT has a strong appeal to common sense that if we are impacting on the global environment then we simply have to reduce population, reduce the economy which causes our over-consumption of the earth, and reduce the technology that is responsible for our impact on the earth.

Unfortunately, IPAT does not leave much hope - for getting a job or reason for having children (both pretty basically dependent on continuing civilization). It can lead to eco-anxiety and even notions of ‘civicide’ [14]. The term ‘eco-anxiety’ was the Oxford word of the year in 2019 but I was aware of its tentacles in the early 1970’s and 1980’s as we began to see the enormity of the issues from global climate change, air pollution, water pollution, loss of biodiversity, and resource constraints like oil. But I could never quite enter into the despair of ‘civicide’ as I kept getting involved in political actions for environmental change and winning! Hope is generated from success in achieving beneficial change as well as from beginning to see a different vision of the future that was emerging, bit by bit [21, 30].

This eventually led me to try and see where we could find a more hopeful model and much of my academic work since has been attempting to show it is possible to find hope in a time of civicide. This work is in finding new technologies, new economic models, new urban planning processes, new politics and new understandings of how we live together on this earth (eg [20, 21, 24, 27, 30, 31, 33–35]). But, before now, I have not related this back to the IPAT model with its powerful arithmetic. So, let me try and do so now.

### Deconstructing the arithmetic in IPAT

It is possible to challenge the arithmetic of IPAT by examining the nature of growth. In this system of thinking:

- Population is seen simply as a multiplying factor on the other two components which are both seen to be inevitably negative.
- Economic activity uses GDP per person to represent how the technology used to create a product is seen to represent consumption of the product thus any economic activity is seen as negative, no matter what it is for; and
- Technological impact is then at the base of the system creating the impact but this is also seen as inevitably negative, as it mostly was at that time when the Ehrlich’s first promulgated IPAT.

Hence the growth in the economy and the increasing number of people inevitably cause all the problems simply by multiplying the impact of growing population based on inevitably negative impacts from all three factors.

It is possible to see how this can be changed as economic activity and technological change are introduced into the system *without having inevitable impact*. The paper will therefore set out three stages in development – exploitive, sustainable and regenerative – that change the IPAT arithmetic.

#### Stage 1 exploitive development

**Fig. 1:**
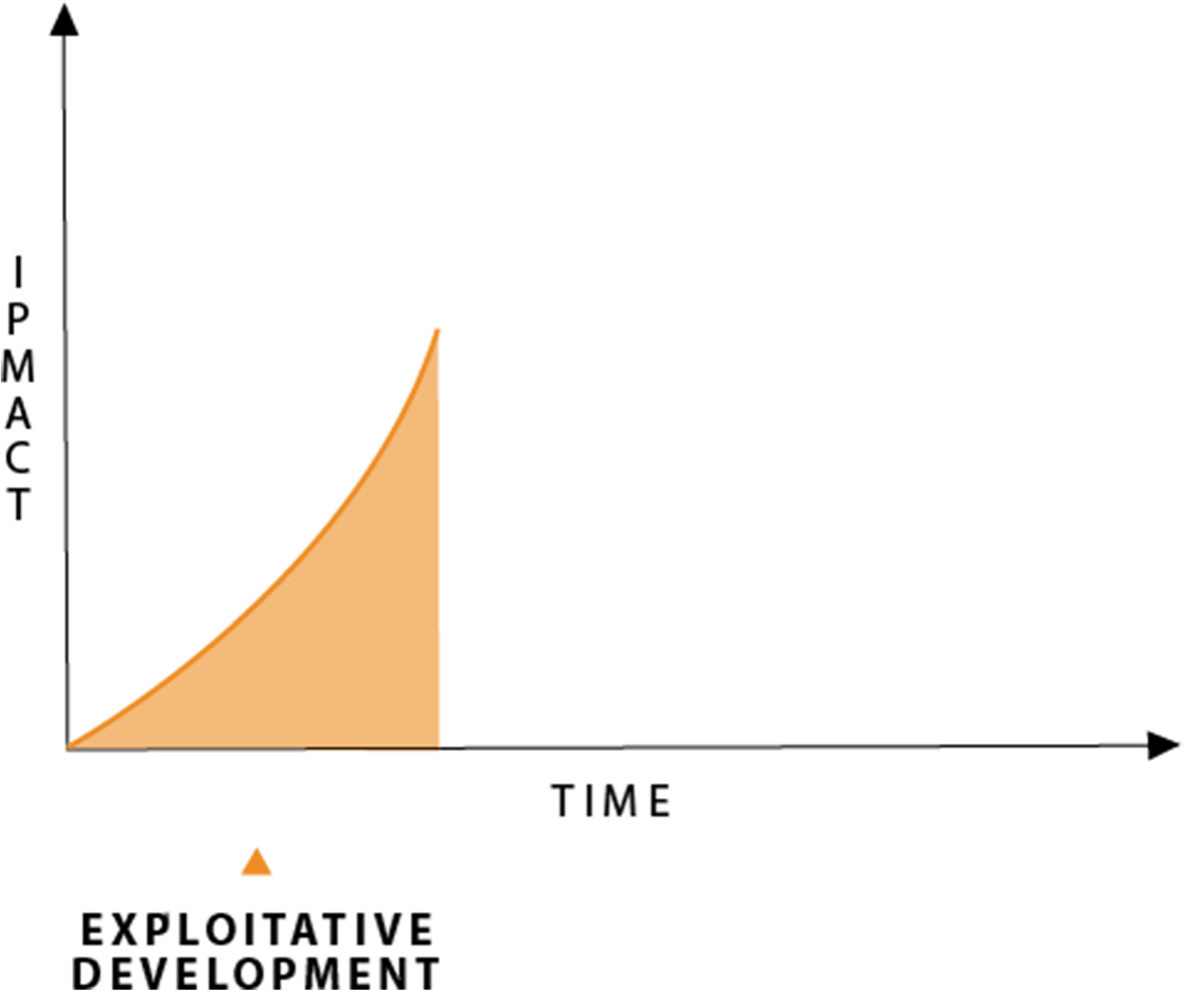
Stage 1, exploitive development

Stage 1 is the exploitive development era, the historic context for the development of IPAT when all three of the components of IPAT appeared to be out of control and were growing exponentially. Figure 1 grows exponentially out of control as all three factors are growing.

However, we have had over 50 years of global action on environmental regulation, environmental technology, environmental assessment and all kinds of environmental governance at global, national and local level driven by strong demand from communities and by some industries. I believe we don’t do justice to this work applying the IPAT model now as the model assumes these factors have not changed and exponential growth in impact will simply continue. Environmental change just disappears under the broad categories of population, affluence and technology, inevitably riding over any reforms. However, these reforms can act on impact without necessarily reducing population or economic growth even within the arithmetic logic of IPAT. But it takes some changes to make the system begin to turn around.

#### Stage 2, sustainable development

If the technology used for creating products and services reduces its impact then the whole system can be improved if it is a big enough change; this is clear in the arithmetic. IPAT reduces impact though it does not mean the overall impact is still not made worse by population and economic growth increases. But it is less impact per unit of population or per unit of economic output and that is a start. I am calling this Stage 2 after getting out of the horrible Stage 1 of exponential growth caused by completely exploitive practices at all levels.

At Stage 2 there is still largely a source of despair as overall impact is still going up even if it has slowed a little. So, people cannot always see how having children could be a hopeful exercise and even economic growth is still largely seen as part of the problem, not part of the solution. This is one of the reasons why there has been a growing division in the world between those who are environmentally-oriented and those who are economically-oriented. It is why the World Commission on Environment and Development was set up and produced the idea of sustainability to try and bring these divisions together, at least in theory [48]. In my reading this has not been related to the arithmetic of IPAT. So, I will show here how it is possible to see how the sustainable development approach works on IPAT if it can be substantial enough:

1. Some people will choose to reduce their impact through all their life choices (so P can be lessened);
2. Some consumption-oriented economic activity can be changed to reduce environmental impact (so A can be lessened); and
3. Some technologies will change to reduce their impact for each product produced (so T is also lessened).

The three reductions *together* combine to make a reduced impact as was clearly the desired outcome from the sustainable development agenda and how this has now led to the Sustainable Development Goals. The three elements of the IPAT equation will mean that there is a system of all three combining to reduce the impact, that would have been, if there had been no change. The idea is that it is *minimized* to enable more social and economic development. That is the fundamental idea behind sustainability. Its overall outcome is designed to flatten the curve of impact.

**Fig. 2:**
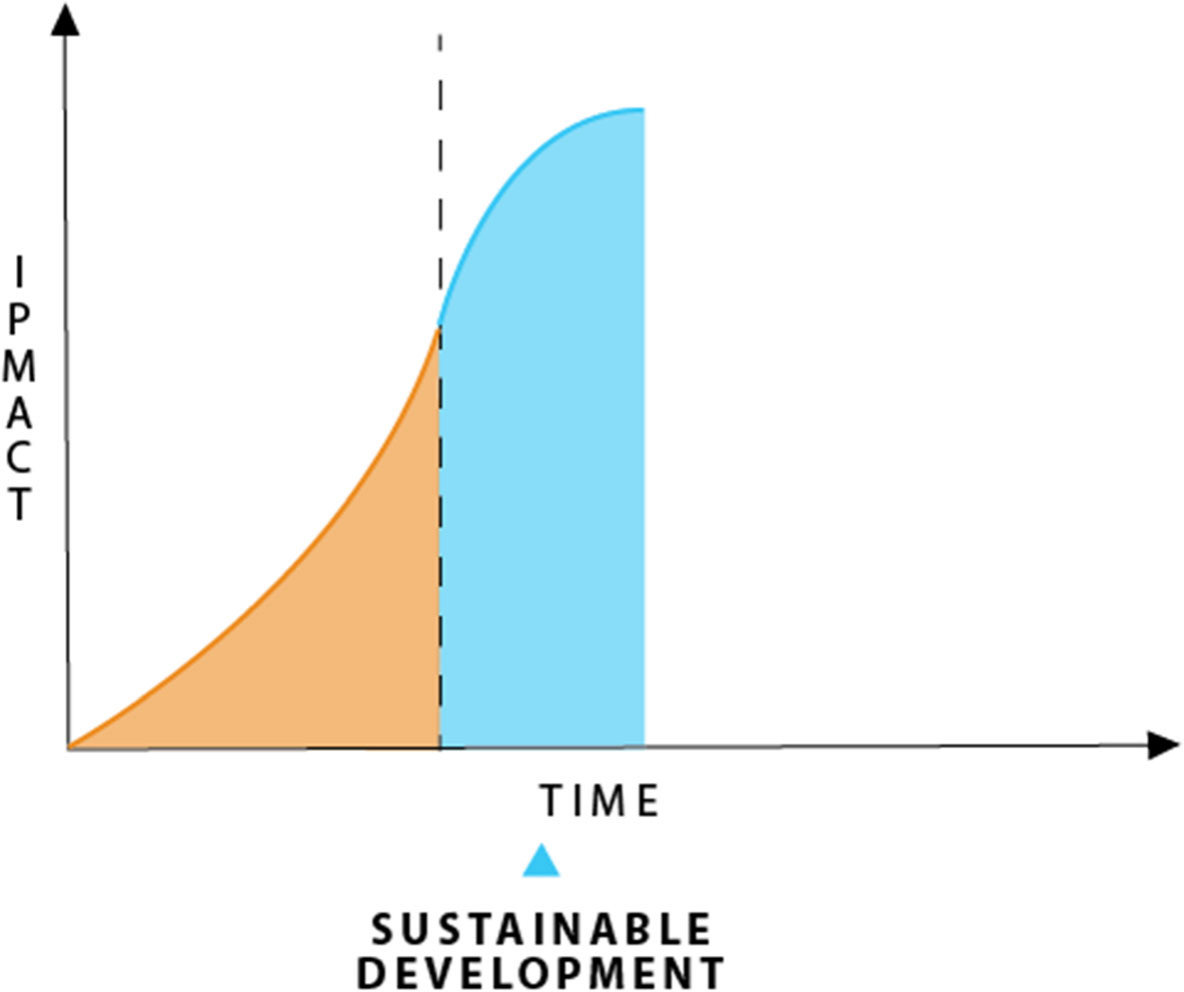
Stage 2 the sustainability phase based on reducing impact

Figure 2 shows how this Stage 2 of Sustainable Development means that the exponentially growing impact begins to slow and eventually reaches a plateau.

**Fig. 3:**
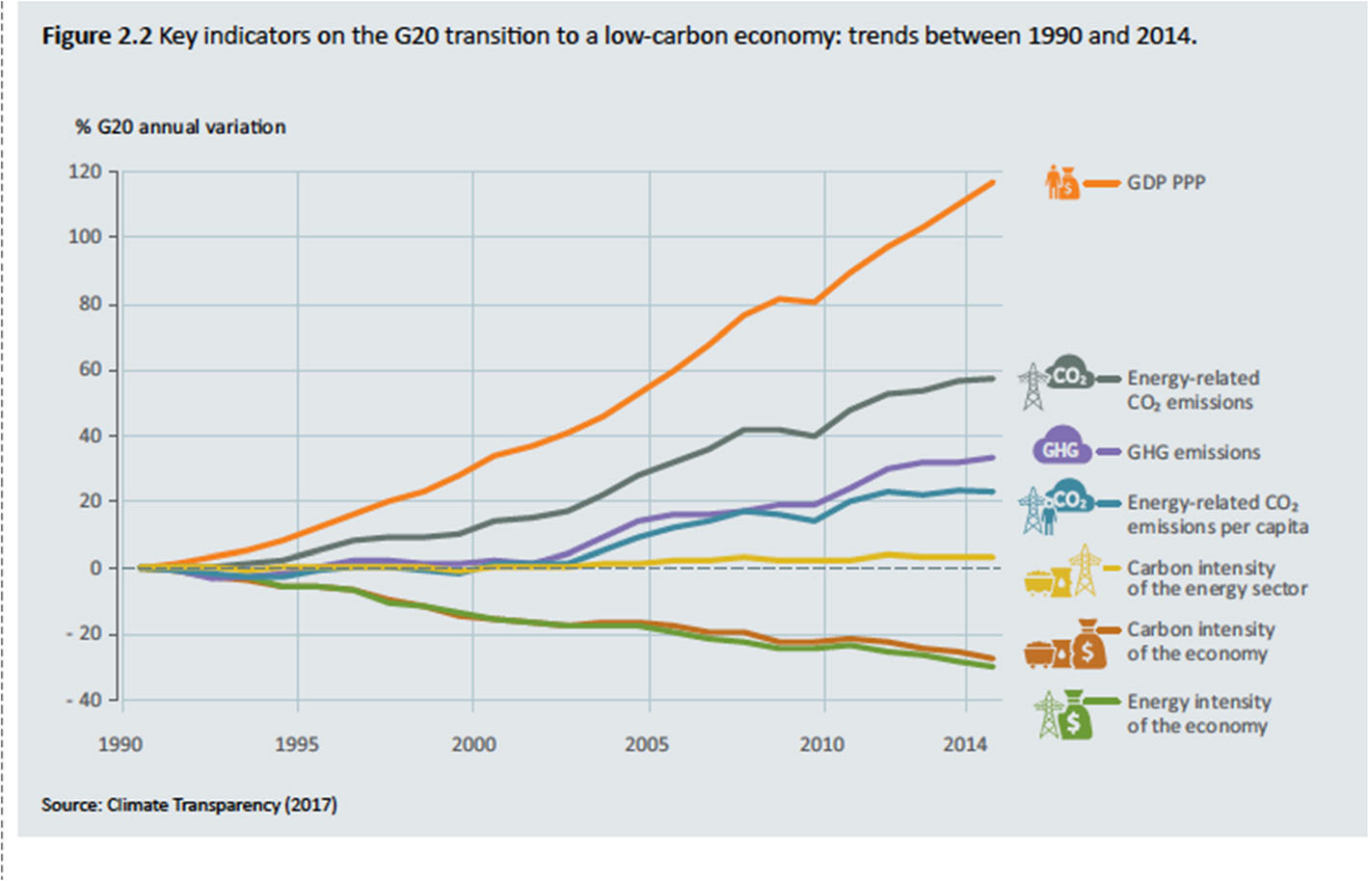
Decoupling impact from economic growth in the G20. Source Climate Transparency (2017)

This Stage 2 of the transition away from global environmental impact is the basis of what is now called Decoupling as shown in Fig. 3 where the G20 nations are summarised in their reductions in impact per unit of population and per unit of economic activity.

**Fig. 4:**
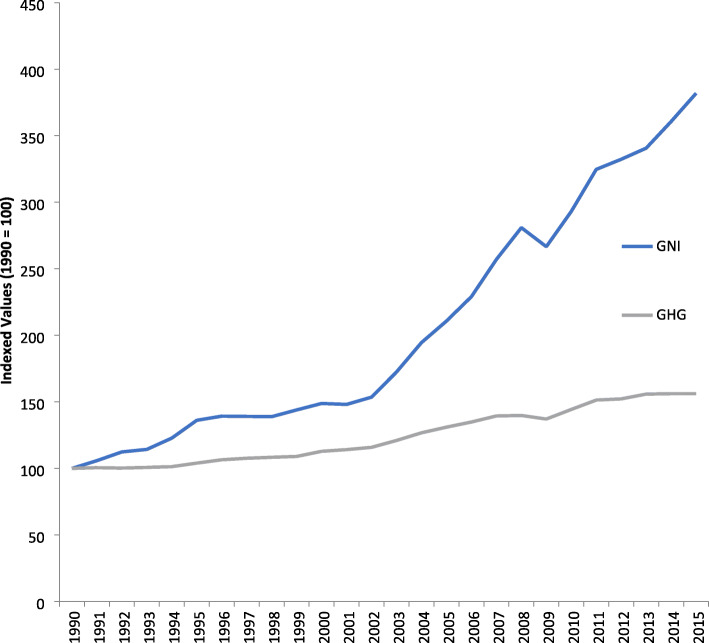
Economic growth per capita vs Greenhouse gas growth per capita, 1990–2015). Source: [28, 36]

Economic growth continues upwards during decoupling but greenhouse gases and other pollution or impacts start reducing and declining. The G20 nations are all decoupling absolutely i.e. they are declining in emissions per capita as per capita GDP goes up ClimateTransparency. Brown to Green Report, Climate Transparency, Berlin, Germany; 2017. We have shown this to be the case in many parts of the world on greenhouse emissions with European nations starting earlier but strong signs now evident in Australia, the US, and China and developing nations like India still increasing emissions and fossil fuel consumption but starting to relatively decouple GHG from economic growth (see [31]; and [28, 36, 46]). So, the overall picture of GDP or GNI vs GHG is now much more positive than it was when IPAT was created (see Fig. 4).

The significance for me here is that there is hope because the changes can happen within:

- the Population factor (people make choices to reduce impact and hence not all population is the problem),
- the Affluence factor (the economic system can be adapted to have lower impact in the process of creating GDP) [18], and
- the Technology factor (the technological system can indeed be a driver of reduced impact).

However, this is still seen by many as being a problem. Despite the rapid change total global impact is still growing despite being much reduced in its growth due to reductions in per capita and per unit of economic activity. Thus, from an IPAT perspective it is not enough as in a finite world it will eventually hit biophysical limits [21, 41]. So, we are still left with the inevitable march towards civicide, though perhaps postponed somewhat.

The reduction of impact can begin small and then grow to be a significant reduction but reduction in per capita is not enough if population is increasing. With the apocalyptic start to 2020 we need to see more or IPAT will keep showing we can never win unless population and economic growth collapse. However, once Stage 2 begins to rollover the top of the curve and start going down, it is a new world that starts unfolding. Stage 2 of the transition begins to absolutely reduce environmental impact in total and offers some hope as it begins to exponentially decrease – just as the problems were exponentially increasing in Stage 1. This is a very significant step and one deserving of hope.

But the big change in understanding IPAT comes when Stage 3 is reached and the system begins to regenerate.

#### Stage 3 regenerative development

If the technology for producing products and services is so changed that it can be regenerative, then we have a totally different perspective on IPAT. Regenerative economic activity and regenerative technology is not just reducing impact it is beginning to regenerate the damaged environment. With regenerative development a whole new system emerges. Using IPAT it is possible to see new opportunities for creating hope in a more sustainable earth.

The IPAT equation in the regenerative Stage 3 is about:

1. Technology that is regenerative, i.e. it is able to not just reduce impact but to regenerate the damage created before. For example, the infrastructure and services of a city could:
  - Make more renewable energy than the city consumes as well as sucking CO_2_ from the atmosphere using carbon absorbing cement, carbon-negative plastics, biogenic building materials, and carbon-negative landscaping [9].
  - Recharge depleted aquifers and rivers.
  - Restore soils and N/P balance in the bioregion as it uses circular economy principles with its waste,
  - Regenerate biodiversity through biophilic habitats on buildings [16, 25, 26, 42]; and
  - All the while growing the community and the economy.
2. Affluence or the consumption of products measured by GDP is now driven by fuels, materials, recyclability, and processes that begin to clean up damaged ecosystems, including the atmosphere with its CO2 no longer growing but declining back to a manageable level.
3. Population now becomes a driving force for regeneration as arithmetically every person added is now using this new economic system and the technological drivers of it, so they will improve the rate of regeneration.

Thus, with regenerative development both population and economic growth can be welcomed as they are driving lots of good. Not just doing less and less bad.

**Fig. 5:**
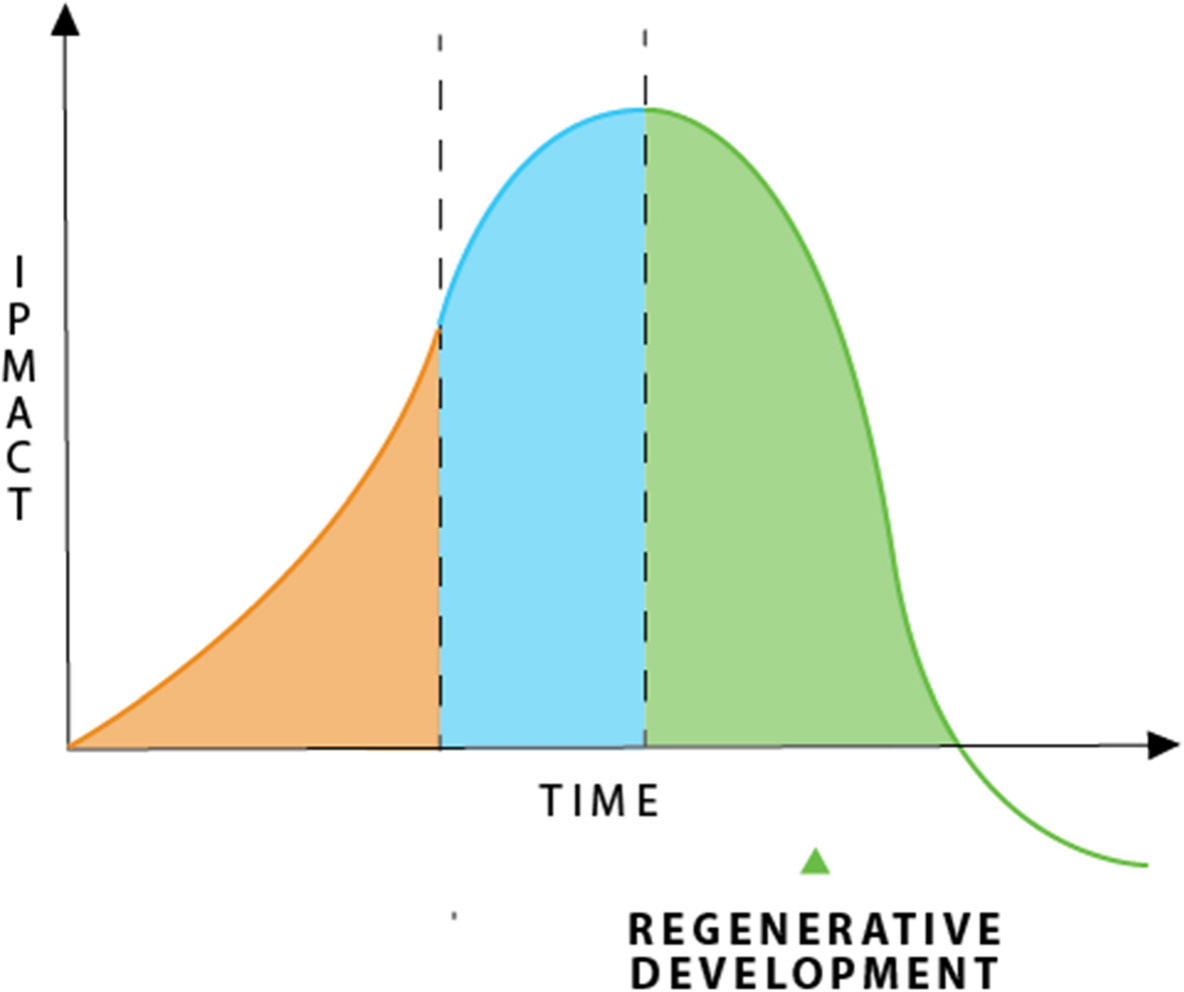
Regenerative development in stage three

This is shown in Fig. 5 where development is regenerating and reclaiming the environment. One word increasingly used in IPCC is transformative [17]. The hope issue then reduces to whether this is likely to happen and how soon. In my world, this is easiest seen through the rapid change in the agenda for cities where regenerative development is replacing sustainable development [16, 43].

## Cities as the basis of reduced and regenerative impact

**Fig. 6:**
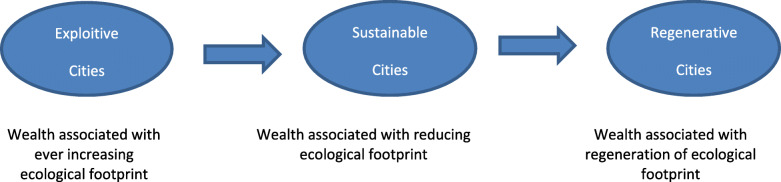
The transition from exploitive to sustainable to regenerative cities. Source Author

The reason why it is important to understand cities as a driving force in the reduced impact economy and the regenerative economy, is that IPAT shows that the integration of people, the economy and technology is in fact a system. In the 1960’s this was out of control and the IPAT analysis helped to drive the changes to create a change to that system. But it is very different when cities begin the transition as shown in Fig. 6 especially when urban planning takes over the agenda from simplistic economic development.

There is a strong case that has been made over many years that cities are a system that can combine these three factors in new ways enabling rapid adjustments to be made [11, 49]. It is not possible to have separated out the population factor from this system as it was a driver in how the economy enabled changes to be made and technologies to be invested. New York is a much cleaner city than it was in the 1960’s and Detroit has changed little as its population declined and its economy stagnated. If IPAT had been right then it could not have explained through simple, linear relationships what are the dynamics leading to New York becoming known as a model for sustainability in the US [31]. There is also no guarantee that simply by growing the population and growing the economy that environmental impact would be reduced, but it is hard to make investments in technological change without growth in these factors. This is the basis of the Environmental Kuznets Curve theory with developing countries and cities [1].

Different parts of cities also lend themselves to different types of change as summarized in Tables 1 and 2 using the Theory of Urban Fabrics [35] and applying it to the urban metabolism in the three types of fabric [43]. Thus creating more walking and transit fabric can enable significantly less urban footprint and make regenerative technology applications much easier [45].

**Table 1:** Resource input variations between urban form types (Source: [43]))

INPUT (Per Person Per Year)	Automobile City	Transit City	Walking City
**Resources**			
Fuel in Megajoules (MJ)^1^	50,000	35,000	20,000
Power in Megajoules (MJ) ^2^	9240	9240	9240
Gas in Megajoules (MJ) ^2^	4900	2940	2940
Total Energy in Gigajoules (GJ)^2^	64.14	47.18	32.18
Water in Kilolitres (Kl)^2^	70	42	35
Food in Kilograms (kg)^3^	451	451	451
Land in Metres Squared (m^2^)^4^	547	214	133
Urban Footprint in Hectares (ha)^5^	2.29	1.97	1.78
**Basic Raw Materials (BRM) for New Building Types Per Person**^**6**^			
BRM 1) Sand in Tonnes (T)	111	73	57
BRM 2) Limestone in Tonnes (T)	67	44	34
BRM 3) Clay in Tonnes (T)	44	29	23
BRM 4) Rock in Tonnes (T)	66	43	33
Total BRM in Tonnes (T)	288	189	147

**Table 2:** Waste output variations between urban form types (Source: [43])

OUTPUT (Per Person Per Year)	Automobile City	Transit City	Walking City
**Waste**			
Greenhouse Gas (Fuel, Power & Gas) in Tonnes (T)^1^	8.01	5.89	4.03
Waste Heat in Gigajoules (GJ)^2^	64.14	47.18	32.18
Sewage (incl. Storm water) in Kilolitres (KL)^3^	80	80	80
Construction & Demolition (C&D) Waste in Tonnes (T)^4^	0.96	0.57	0.38
Household Waste in Tonnes (T)^5^	0.63	0.56	0.49

The next phase of regenerative impact will be powerfully led by cities as they have the resources, the growth and the innovative capacity to make such changes. We have set out some of the emerging regenerative technologies [43, 44] and how they can help shape cities [30]).

**Fig. 7:**
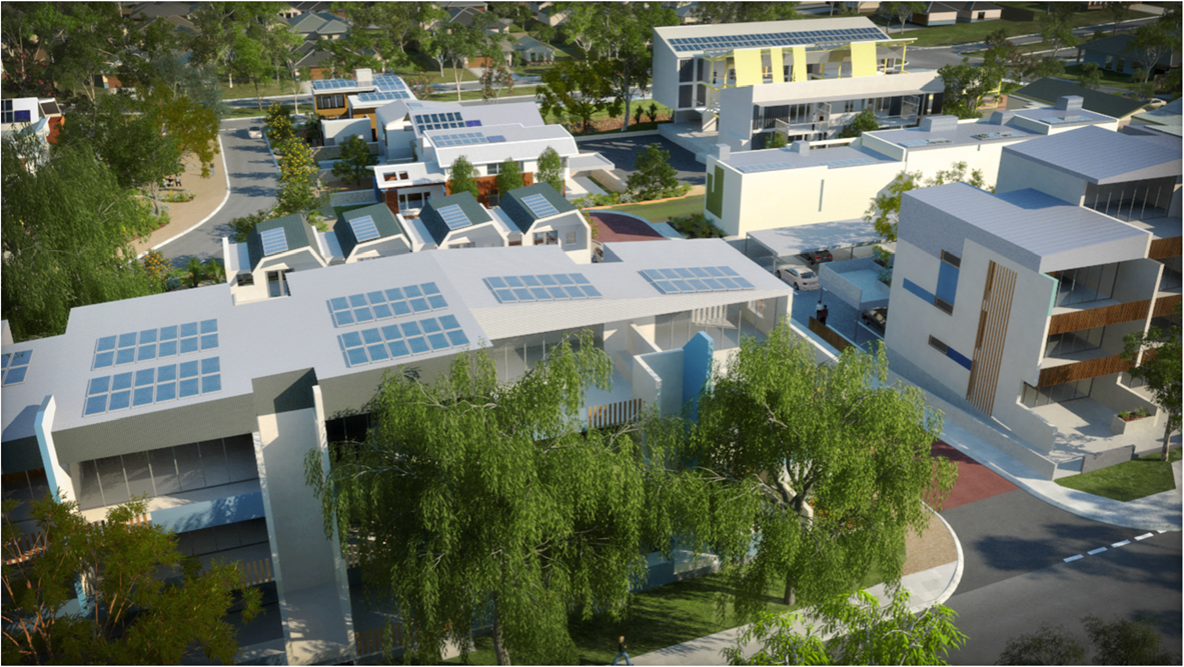
WGV in Fremantle, Australia produces more solar energy than it consumes and has created new ways of sharing solar using Blockchain

The growth in solar, batteries and electric vehicles promises a way for cities to export renewable energy to their regions rather than the reverse [6, 13, 40]. WGV in Perth is a development generated as a model for how this can be done [50] – see Fig. 7.

**Fig. 8:**
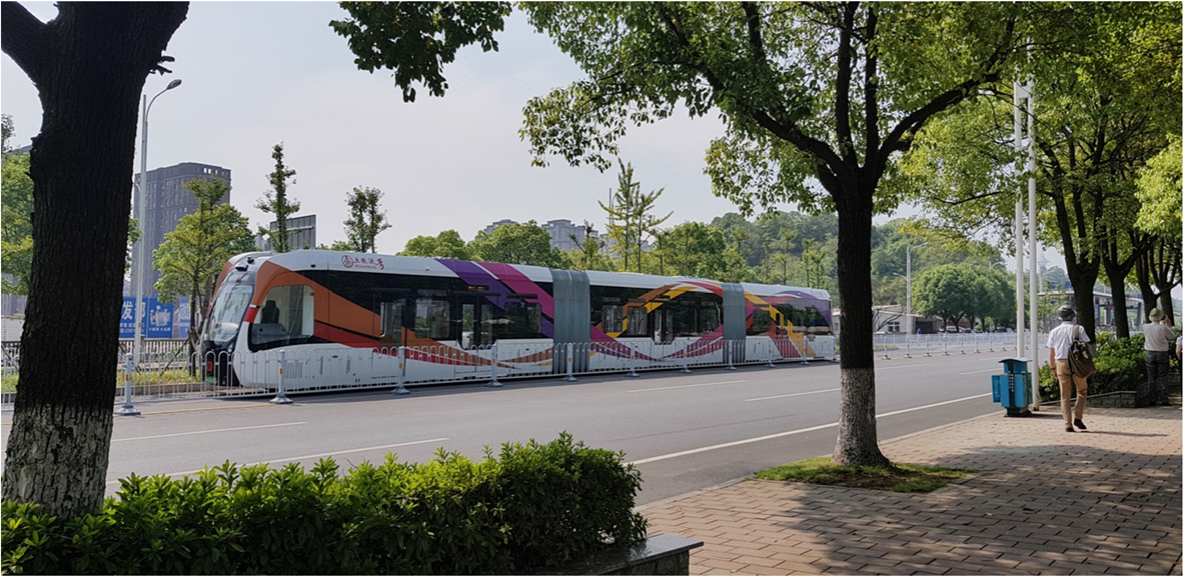
A Chinese Trackless Tram in Zhu Zhou

At the same time new signs of hope are appearing in cities as they come to terms with reduced growth in automobile dependence [35]. The peak car phenomenon has shown that cities can decouple GDP and car use and this phenomenon can now be seen in Shanghai and Beijing [10]. New electric Trackless Trams have appeared (Fig. 8) as a dramatic way to both provide a far superior kind of mobility down a main road corridor but also to create a Transit Activated Corridor of urban regeneration [32, 37, 38].

### The transition to regenerative impacts

**Fig. 9:**
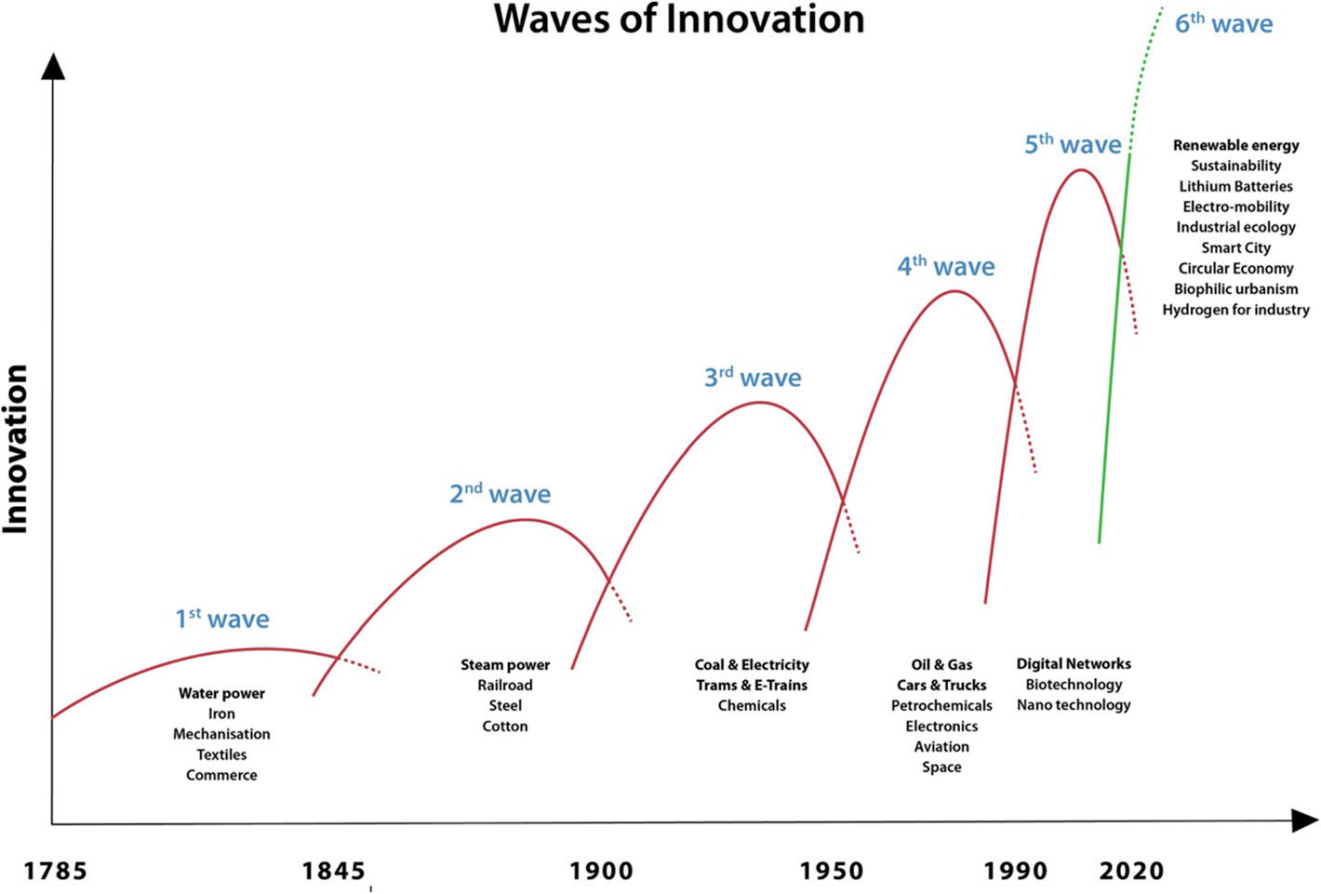
Innovation waves and transitions to new economic systems. Source [15]

[15] set out, quite prophetically, what could be expected in the transition to new technologies and urban systems after the next big crash (Fig. 9). This was based on Kondratiev Curves that show how economies change drastically as they come out of major economic decline as happened in the 1840’s, 1890’s, 1930’s, 1980’s and now is likely in the 2020’s especially following the Covid collapse.

The 6th wave, as seen by [15] suggests we could anticipate to build on the digital wave into a future based on sustainability-based technologies and systems which were emerging at that time in 2006. Perhaps we can now see that it is going to be based on a range of technologies that are likely to go beyond sustainable development to being regenerative development. Hargroves and his team [15] re-examined his earlier work in von Weisaker et al., 2009 [47] and suggested even stronger potential for transformative change.

**Fig. 10:**
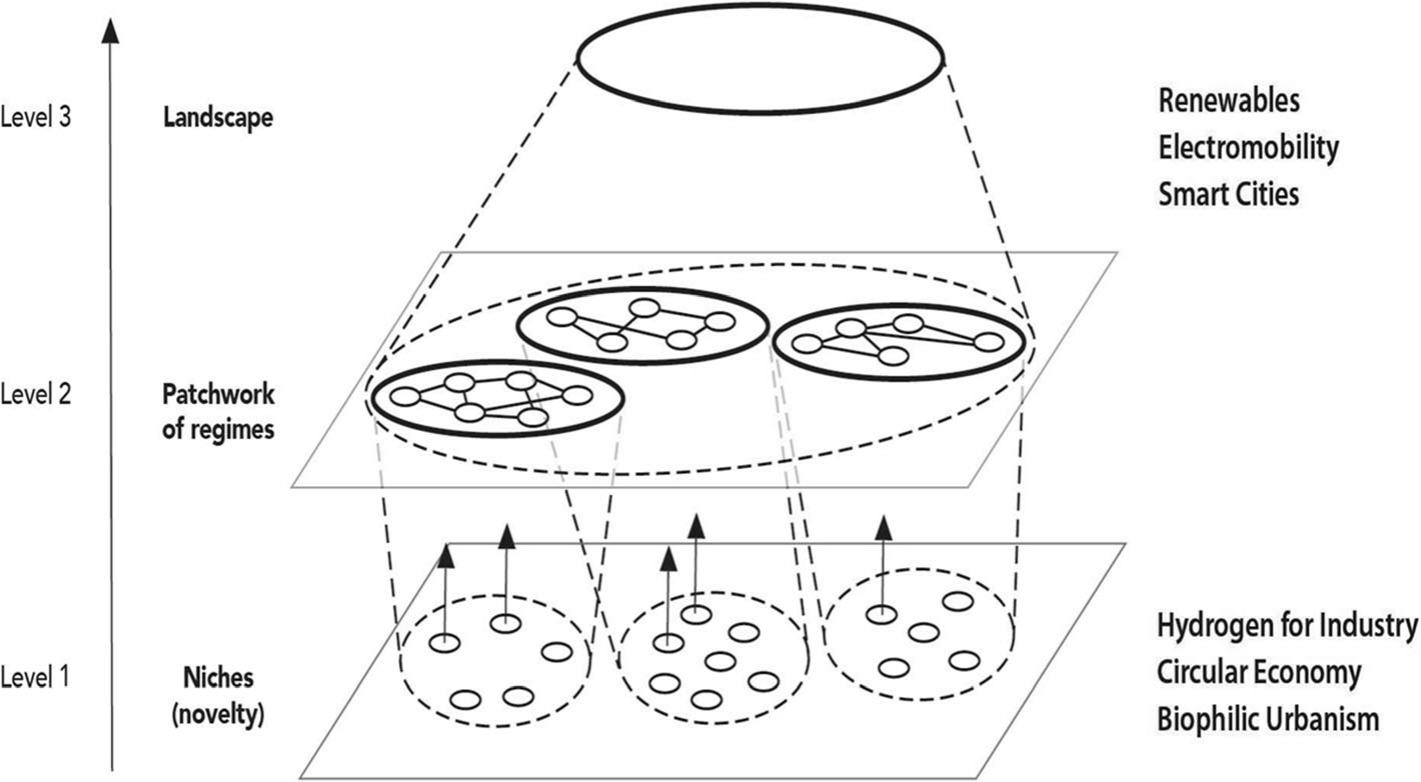
The multi-level perspective of Transition Theory showing how technologies niches to regimes to mainstream landscapes [12, 37]

The need for technology to be mainstreamed using the socio-technical systems set up through government regulation, cultural choices and professional practices, is the basis of Transition Theory as set out by Geels et al. [12] and illustrated in Fig. 10.

The solar-batteries-EV’s-smart city technologies are all rapidly mainstreaming while hydrogen, circular economy and biophilic urbanism are still needing more R&D and demonstrations. These are all potentially regenerative-based technologies and systems and in the next decade we may begin to see some cities moving to create whole corridors of urban regenerative development and eventually across the whole urban area. This will mean we can look back at the three stages of impact reduction from the exploitive to the sustainable to the regenerative as in Fig. 5 and see transformative change. Hope is believing that this can occur fast enough to avoid civicide.

## The Hope transition fundamentals

The grasping of hope in the midst of civicidal fear and despair is a spiritual journey that has been a societal issue for 5000 years. The writing in this space is labelled apocalypse literature and is about cataclysmic change symbolising the end times. It has been a part of Jewish, Muslim and Christian literature [2, 4] and related to: pandemics, famine from climate change, war (continuous conflict) and death (of civilization). Such ideas have been used in the past two thousand years to portray starkly different futures through art and literature [3, 39]. In the Christian tradition the author of the last book of the bible was writing in the time of the collapse of the Roman Empire. The future was portrayed as a cataclysmic choice between two cities that were based either on frivolous consumption that would collapse or was based on long term meaningful work, represented by building the city from diamonds, in harmony with the Tree of Life and River of Life [30]. Seeking to find regenerative diamonds to build the city of the future is the basis of my hope and I believe we have no choice but to move firmly in this direction [29]. If we do not then the apocalypse of IPAT, which Hancock calls civicide, will happen.

## Conclusions

There is hope in a time of civicide as we have moved a long way from the 1970’s and 80’s where IPAT set out a troubling future if we did not change. Much has changed and we have new socio-technical systems that are significantly more sustainable and even regenerative, so we can now move these towards being mainstreamed. But we have to choose this future and not go back to the exploitive approach to development so embedded at that time.

This paper suggests that we can ‘practice’ in ways that enable the next economic cycle to be one that is based on moving from sustainable to regenerative technology and their associated socio-technical systems. The vision and developing practice can restore hope and prevent us from needing to praise civicide as the only way to solve global environmental problems.

In this perspective the pandemic’s impact is likely to be seen in the future as relatively positive on the development of a future economy. It will show that significant and deep issues like climate change required cities and nations to stop doing what they had become fixated to do and rapidly phased out fossil fuels.

IPAT has continued to explain a despairing future when the exploitive approach to development was the only way to the future. This meant we were locked into social and economic systems, in regulatory and financial systems, that had to be broken down before they could be built up in the new ways. The pandemic has broken much of this down and we have learned now how we can go beyond sustainable to regenerative development. At least we can begin to see where it is possible and we need to grasp every opportunity now to show this is the way to prevent civicide.

## Data Availability

There is no data or material to add.
